# Sonographic exploration for fascial exploration (SEFE) in necrotizing fasciitis: a case report

**DOI:** 10.1186/s13089-020-00168-5

**Published:** 2020-04-22

**Authors:** Jessica Fozard, Krystle Shafer, Thompson Kehrl

**Affiliations:** WellSpan York Hospital Emergency Medicine Residency Program, WellSpan York Hospital, York, PA USA

**Keywords:** Point-of-care ultrasound, Necrotizing fasciitis, Musculoskeletal ultrasound

## Abstract

**Background:**

Necrotizing skin and soft tissue infections are life-threatening conditions. Reliance on gas in tissue planes leads to worsened outcomes in patients with non-gas forming types of necrotizing fasciitis (NF).

**Case presentation:**

We present a case of Group A Strep (GAS) necrotizing fasciitis, which was identified at bedside with point-of-care ultrasound (US) including an area of subfascial fluid. Computerized tomography only revealed diffuse cellulitic changes. Patient was taken to the operating room where fascial exploration was not performed at the concerning area seen on ultrasound and thus falsely negative. The patient subsequently developed multi-system organ failure and required amputation of the limb due to rapid spread of GAS NF.

**Conclusion:**

We suggest an US protocol to help identify optimal areas for fascial exploration—sonographic exploration for fascial exploration (SEFE).

## Background

Utilization of CT scan for diagnosis of NF is supported only by small studies that which focus on images using IV contrast enhancement. The most common CT finding is focal subcutaneous fat infiltration and fascial thickening [1]. These findings lack specificity and can be seen with non-necrotizing infections and even non-inflammatory conditions [2]. MRI is considered to be the current gold standard imaging testing, with a sensitivity of 93% in detecting NF [3]. This imaging modality, however, can be time consuming to obtain in a rapidly fatal disease. With regard to US, there are emerging research and approaches for the diagnosis of necrotizing soft tissue infections as there have been multiple case reports that demonstrated accurate diagnosis of NF with US where as CT and MR only demonstrated changes consistent with cellulitis [4]. The STAFF exam provides a simple and memorable acronym for identifiable signs of necrotizing soft tissue infection (NSTI) on US—subcutaneous thickening, air, and fascial fluid [5]. With regard to fascial fluid amount, the best cut-off to diagnose NF is reported as 2 mm of fluid accumulation, which has a sensitivity of 75% and a specificity of 70.2% [6]. However, data with US is limited as well, and a negative US exam, much like a negative CT scan or LRINEC (laboratory risk indicator for necrotizing fasciitis) score, does not exclude the diagnosis [5]. US is reported to have a sensitivity of 88.2%, specificity of 93.3%, a positive predicative value of 83.3%, a negative predictive value of 95.4% and an accuracy of 91.9% in the diagnosis of NF [7]. Surgical exploration remains the gold standard for both diagnosis and treatment and was appropriately performed in this patient case [8]. The time to successful surgical debridement is considered the single most important variable influencing mortality in this patient population [9]. What is unique about our case goes beyond using US for diagnosis, but also using this modality to help identify the muscle compartment to be explored during surgery. If the US results were utilized in this case to aid the site of surgical exploration, the ultimate outcome of this patient may have been different.

With regard to the surgical decisions on areas of debridement for NF, examination of surgical reference textbooks reveals vague allusions to “immediate and extensive surgical debridement”, but without any specific technique mentioned [10, 11]. A surgical review article on NF states to make an incision over any “overtly necrotic area”, and when this is not evident, to make an incision over the area “deemed to be the center of the disease process”, without any further elaboration [12]. This lack of standardization is particularly concerning in early NSTIs when the skin may be spared and not exhibit any impressive or obvious appearance of underlying pathology. Our patient in this case is an example of this, as her skin changes were misleading in the operating room (her entire left lower extremity was mottled) and led to two negative biopsies. The specific location of the infection was correctly identified on bedside ultrasound and but not on CT. Unfortunately, the compartment identified on ultrasound was not explored during the initial operation.

The utility of US to help guide surgical debridement with regard to the extent or timeliness has yet to be defined in the literature. In this particular case described below, US would have been helpful in guiding the surgeons toward a specific anatomic location for their surgical incision and drainage. Other than the STAFF exam, there does not appear to be any unified or systematic process for evaluating a limb or area for underlying NF when the area involved is either extensive or uncertain. In a review of the US literature, we found a routine approach to soft tissue ultrasound that could be applied by trained physicians to systematically evaluate an extremity. The same principles could be applied to other areas of the body as well. Primarily, scanning an unaffected area/contralateral extremity, as well as over areas that are concerning for pathology [13] should be considered. Scanning in multiple planes is also recommended for localization and providing as much information as possible [13]. We propose combining both approaches to create a protocolized US to help identify optimal areas for fascial exploration—sonographic exploration for fascial exploration (SEFE) (Table 1).

**Table 1:** Explanation of the steps to perform a SEFE examination

SEFE examination
Step 1: Scan all fascial compartments (such as anterior, lateral, superficial posterior and deep posterior in the lower extremity) even if skin changes are not present
Step 2: Do you have BOTH diffuse subcutaneous thickening AND fascial fluid > 2 mm present? = If so, this is diagnostic for NF
Step 3: Additionally look for supporting, but not mandated findings such as subcutaneous air or abnormal architecture of the muscle tissue
Step 4: Mark area of US findings on patient skin and consult surgery for exploration

## Case presentation

**Fig. 1:**
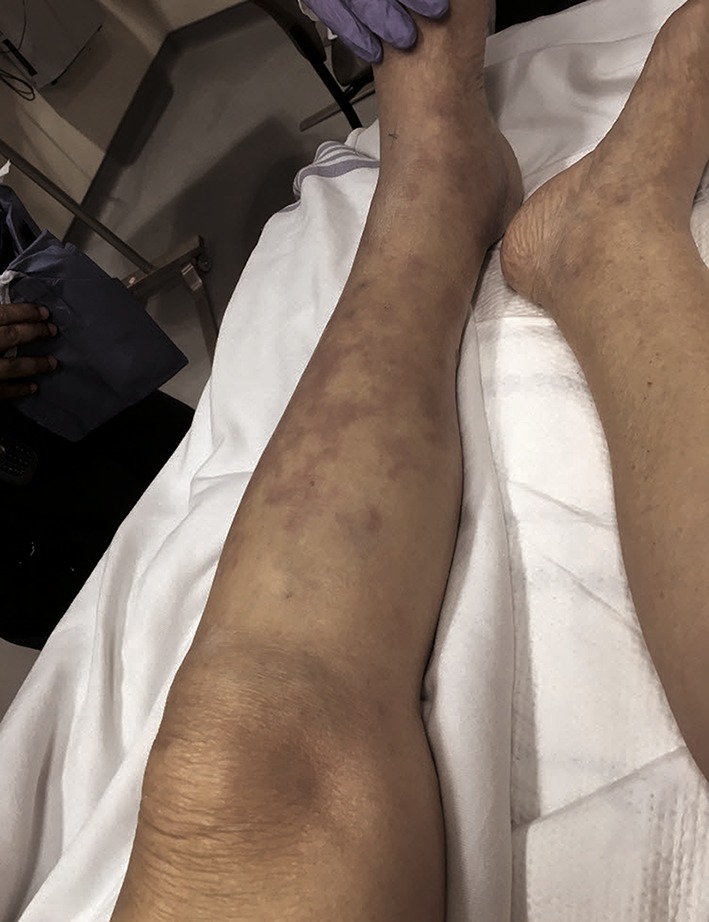
Left lower extremity upon presentation to the emergency department. Note mottling of skin but no bullae

An 80-year-old female presented to the Emergency Department (ED) via ambulance with altered mental status. Her family reported the patient developed rapid onset of and worsening lethargy that started shortly after she awoke in the morning. Emergency medical services (EMS) noted the patient to be tachycardic and hypotensive en route. Upon arrival to the ED, she was somnolent but arousable to voice and unable to provide any details. Her past medical history included diabetes and hypertension. Vital signs upon presentation included a temperature of 38.4 °C, heart rate 128 beats per minute, blood pressure 100/64 mm Hg, respiratory rate 32, and pulse oximetry of 97% on room air. On exam she was noted to have a mottled left lower extremity (Fig. 1) with intact distal dorsal pedal and posterior tibial pulses. No crepitus or bullae were noted. Palpation of her entire left lower extremity appeared to be painful, especially in her groin and proximal calf area.

**Fig. 2:**
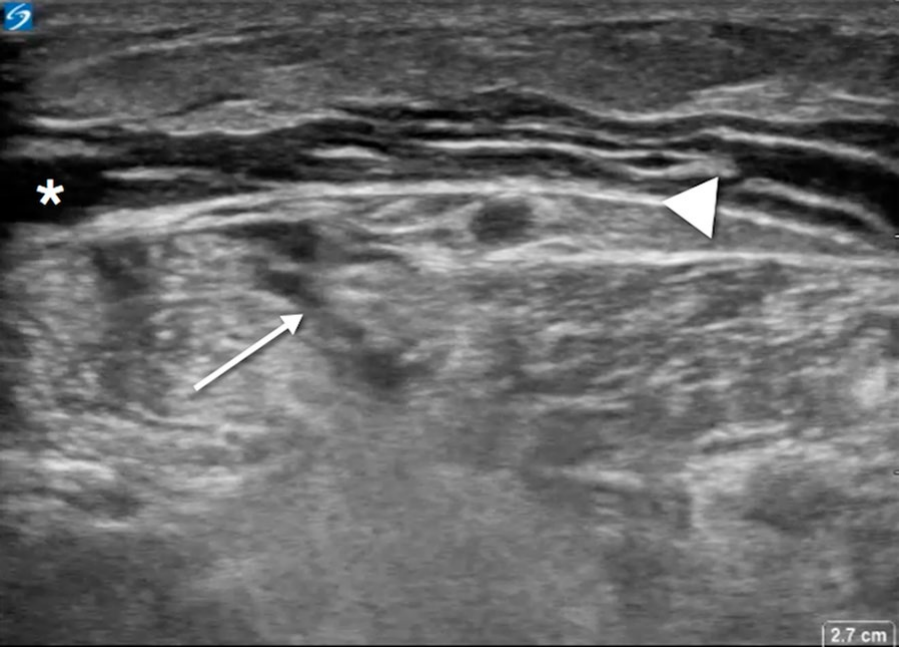
Transverse view of posterior left lower extremity distal to popliteal crease. Fascia is indicated by arrowhead. Anechoic fluid collection is noted anterior to fascia (*). Deep to fascial layer, a complex fluid collection is noted (arrow) with diffuse hyperechogenicity of gastrocnemius

Laboratory analysis included white blood cell count of 12.2 and a significantly elevated venous lactic acid at 9.8. Acute renal failure was noted with a serum creatinine of 3.05 (baseline 1.00). Sodium was 140 and glucose 167. A venous blood gas showed pH 7.21, pCO_2_ 32, and HCO3 13. Blood cultures were sent. Left lower leg X-rays showed mild soft tissue edema, but no gas in tissue planes. A point-of-care ultrasound (US) was performed (SonoSite, Bothell, WA) and showed thickened subcutaneous tissue with fluid accumulating superficial to the deep fascial layer > 4 mm in the superficial posterior compartment of the left lower leg. Distal to the popliteal fossa on the posterior aspect of the left leg deep to the fascial layer there was an anechoic fluid pocket with swirling of fluid noted with transducer compression (Additional file 1: Video 1). Abnormal sonographic architecture of the gastrocnemius muscle was noted as well (Fig. 2). Additional file 2: Video 2 displays a normal lower extremity ultrasound for comparison purposes.

**Fig. 3:**
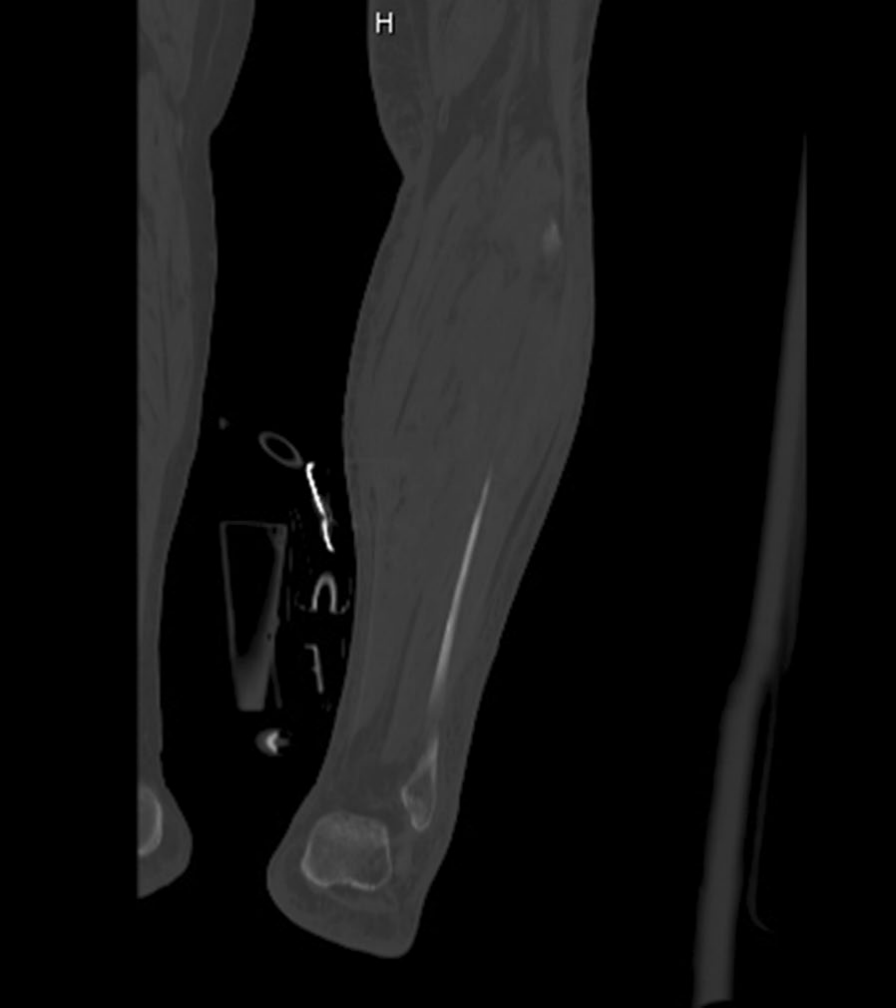
CT sagittal view of the left lower extremity which reveals mild diffuse fatty stranding

Patient was diagnosed with necrotizing fasciitis and ordered broad-spectrum antibiotics: vancomycin, clindamycin, and piperacillin–tazobactam. She was resuscitated with intravenous fluids. Surgical consultation was obtained. Recommendations included computerized tomography (CT) of the left lower extremity of which was obtained (Fig. 3). This was performed without intravenous contrast and showed soft tissue swelling, subcutaneous fatty standing and mildly enlarged lymph nodes in the left inguinal region with possible small hematoma. The radiologist’s differential diagnosis included cellulitis or anasarca. Diffuse subcutaneous fatty stranding was noted in the left calf, ankle and foot region, with edema being most likely.

She was taken to the operating room by the surgical service where two incisions were made. The first surgical incision was made on the left lateral leg just anterior to the greater trochanter and was taken down to the level of the fascia. Fascial planes appeared intact and there was no evidence of necrotizing process. A second incision was made just inferior to the head of the fibula in the lateral compartment and again taken down to the level of the fascia, which was not entered. Intra-operative compartment pressures were measured and noted to be normal. The left lower leg incision was closed with staples and a vacuum dressing was placed on the left thigh incision.

Post-operatively, her clinical condition worsened. Both blood and intra-operative cultures grew Group A *Streptococcus*. She returned to the operating room on hospital day #2. Her lateral calf incision was extended proximally and distally; the fascia was opened up in all four lower extremity compartments and necrotic tissue was noted diffusely. Because of extent of necrosis in the left lower extremity, a below the knee guillotine amputation was performed. She returned to the operating room on hospital day #3 for further extensive debridement. On hospital day #4 her condition continued to worsen and she expired.

## Conclusions

When the diagnosis of necrotizing fasciitis is made and involves a location with multiple potential muscle compartments as potential etiologies (such as the lower extremity) and CT/X-ray imaging does not reveal obvious location, we recommend the following approach: using the distal lower extremity as the example, we recommend scanning all four fascial compartments (anterior, lateral, superficial posterior and deep posterior) regardless of skin findings on clinical exam. A linear transducer should be utilized and each compartment should be scanned in multiple planes. Specific attention should be paid attention to subcutaneous tissue and fascial planes. As identified by the STAFF exam, the examiner should look for subcutaneous thickening, air, and fascial fluid. Additional findings may include abnormal architecture of the muscle tissue as was identified in our patient case. Applying this technique has the potential to serve as both an additional diagnostic tool and as guidance for successful operative exploration and debridement. However, it should be noted that more data are needed on this topic such as a prospective clinical trial and ultrasound should never be used as a rule-out test. While we should use every tool at our disposal to diagnose this rapidly fatal condition and point-of-care ultrasound findings can be integrated with the clinical picture, it still remains that surgical exploration is the gold standard for diagnosis of NF.

## Supplementary information

**Additional file 1: Video 1.** Transverse view of the patient’s posterior left lower extremity in same anatomic site as Fig. 2. Note swirling of complex fluid deep to fascia

**Additional file 2: Video 2.** Transverse view of a normal posterior left lower extremity in same anatomic site as Fig. 2.

## Data Availability

There is no additional data or material.

## Abbreviations

NF: Necrotizing fasciitis
GAS: Group A Strep
US: Ultrasound
SEFE: Sonographic exploration for fascial exploration
ED: Emergency Department
EMS: Emergency medical services
CT: Computerized tomography
NSTI: Necrotizing soft tissue infection
LRINEC: Laboratory risk indicator for necrotizing fasciitis

## References

[1] Hayeri M, Ziai P, Shehata M, Teytelboym O, Huang B (2016). Soft-tissue infection and their imaging mimics: from cellulitis to necrotizing fascitis. Radiographics.

[2] Malghem J, Lecouvet F, Omoumi P, Maldague B, Vande Berg B (2013). Necrotizing fascitis: contribution and limitations of diagnostic imaging. Joint Bone Spine.

[3] Ali S, Srinivasan S, Peh W (2014). MRI in necrotizing fasciitis of the extremities. Br J Radiol.

[4] Misiakos E, Bagias G, Patapis P, Sotiropoulos D, Kanavidis P, Machairas A (2014). Current concepts in the management of necrotizing fasciitis. Front Surg.

[5] Castleberg E, Jenson N, Dinh V (2014). Diagnosis of necrotizing fasciitis with bedside ultrasound: the STAFF exam. West J Emerg Med.

[6] Lin C, Hsiao C, Chang C, Huang T, Hsiao K, Chen Y, Fann W (2019). The relationship between fluid accumulation in ultrasonography and the diagnosis and prognosis of patients with necrotizing fasciitis. Ultrasound Med Biol.

[7] Yen Z, Wang H, Ma H, Chen S, Chen W (2002). Ultrasonographic screening of clinically-suspected necrotizing fasciitis. Acad Emerg Med.

[8] Bonne S, Kadri S (2017). Evaluation and management of necrotizing soft tissue infections. Infec Dis Clin North Am.

[9] Bellapianta J, Ljungquist K, Tobin E, Uhl R (2009). Necrotizing fasciitis. J Am Acad Orthop Surg.

[10] Marcucci L, Moritz MJ, Chen H (2006). Avoiding common surgical errors.

[11] Michael WM et al; illustrations by Holly R. Fischer. (2006). Greenfield’s surgery: scientific principles and practice. Philadelphia: Lippincott Williams & Wilkins

[12] Hakkarainen T, Kopari N, Pham T, Evans H (2014). Necrotizing soft tissue infections: review and current concepts in treatment, systems of care, and outcomes. Curr Probl Surg.

[13] Kaplan P, Matamoros A, Anderson J (1990). Sonography of the musculoskeletal system. Am J Roentgenol.

